# Pharmacokinetic Study of 7 Compounds Following Oral Administration of Fructus Aurantii to Depressive Rats

**DOI:** 10.3389/fphar.2018.00131

**Published:** 2018-03-05

**Authors:** Xianhua Zhang, Linran Han, Jin Liu, Qiuyue Xu, Yuxin Guo, Wan Zheng, Jian Wang, Xi Huang, Ping Ren

**Affiliations:** ^1^Institute of TCM-Related Comorbid Depression, Nanjing University of Chinese Medicine, Nanjing, China; ^2^Department of Pharmacy, Xiamen University Hospital, Xiamen, China; ^3^Department of Clinical Pharmacology, Xiangya Hospital, Central South University, Changsha, China; ^4^Hunan Key Laboratory of Pharmacogenetics, Institute of Clinical Pharmacology, Central South University, Changsha, China; ^5^Mental Health Institute, The Second Xiangya Hospital of Central South University, Changsha, China; ^6^Clinical Pharmaceutics Room, Shenzhen Children’s Hospital, Shenzhen, China; ^7^Department of Human Anatomy, Basic Medical College of Zhengzhou University, Zhengzhou, China; ^8^Department of Anesthesiology and Critical Care Medicine, Johns Hopkins University School of Medicine, Baltimore, MD, United States

**Keywords:** Fructus Aurantii, herb, pharmacokinetic, LC/MS/MS, anti-depression

## Abstract

In the present study, the pharmacokinetics of multi-components (naringenin, nobiletin, meranzin hydrate, narirutin, naringin, hesperidin, and neohesperidin) were investigated in acute depressive rats following oral administration of Fructus Aurantii (Zhi-Qiao, ZQ) extract (20 g/kg). A rapid and reliable liquid chromatography-tandem mass spectrometry (LC-MS/MS) method was established to quantitatively or qualitatively analyze the 7 absorbed ingredients in the plasma, hippocampus and cortex of acute depressive rats. Biological samples were separated on a 300SB-C18 column, and the 7 compounds were detected with sequential positive and negative ionization modes. Our results confirmed that ZQ has antidepressant effects by decreasing the immobility time. In addition, this validated method showed good linearity (*r* ≥ 0.9987), and the lower limits of quantification were 2.73–16.38 ng/mL for the 7 analytes. This method successfully determined the pharmacokinetics of the 7 compounds and separated two pairs of isomers in plasma of acute depressive rats following oral administration of ZQ extracts. The 7 active ingredients were also identified as marked compounds in target tissues and should be further examined in pharmacokinetic studies with acute depressive rats. So, pharmacokinetic compounds were precisely linked with the antidepressant effect of ZQ in our study. This relationship is well-understood and contributes to the application of Traditional Chinese Medicine (TCM).

## Introduction

Depression is a common mental disorder, with the main clinical features of atypical behaviors, low mood and loss of interest (Rothkirch et al., 2017). This disease may increase risk for disability and suicidality, and impose a substantial burden on patients, their kinsfolk, and entire community (Simon, 2003). It is reported that about 10% of the world population was affected by depression, with a lifetime prevalence of 7–21% (Flores-Burgess et al., 2017; Liu et al., 2017). Several studies showed a strong association between stress exposure and various psychiatric disorders especially depression (Kendler et al., 2001; Budni et al., 2013). Stressful life events such as gender-based violence, sudden loss of beloved, chronic diseases, and poverty, have been deemed to play a crucial role in the course of depression (Amiri et al., 2015). Meanwhile, it has been indicated that *N*-methyl-D-aspartic acid (NMDA) receptors’ activation induces negative neural alteration, leading to behaviors relevant to depression under stressful conditions (Haj-Mirzaian et al., 2015). Furthermore, several evidences also documented that selective serotonin reuptake inhibitor (SSRI) could decrease NMDA receptors’ activity (Haj-Mirzaian et al., 2016). A serotonin-norepinephrine reuptake inhibitor (SNRI) or SSRI is widely used as a first-line therapeutic, but 55–81% older persons with major depressive disorder (MDD) fail to recover due to treatment resistance (Lenze et al., 2015) and SSRIs also had to discontinue because of inhibiting gut motor via 5-HT_2_ receptor and acyl ghrelin (Fujitsuka et al., 2009). The former constitutes common and potentially life threatening in aged people with MDD, but clinicians know little about the risks of augmentation of pharmacotherapy (Schneider et al., 2006), and the latter incidence is high. However, based on 1000s of years of experience, Traditional Chinese Medicine (TCM) is safe and focuses on a “holistic” approach. For example, Fructus Aurantii, which is the dried and closely mature fruit of *Citrus aurantium L*., is one of the most popular traditional herbal medicines that has anti-oxidant (Anagnostopoulou et al., 2005), anti-tumor (Miki et al., 2002), anti-hypertension (Yanez et al., 2004), and anti-inflammatory effects (Wu et al., 2006; Huang et al., 2011). Fructus Aurantii is also called Zhi-Qiao (ZQ) in China, and mainly includes flavonoid glycosides and polymethoxylated flavones. Studies have shown that ZQ possesses prokinetic and anti-dyspepsia functions (Wang et al., 2015; Xiong et al., 2015) different from SSRIs. On behalf of “disperse stagnated liver and qi stagnation” in Traditional Chinese Medicine, ZQ has been commonly utilized for remitting depression-like symptoms such as pain, insomnia, functional dyspepsia and depression for approximately 2000 years (Zhang Y.J. et al., 2012).

The key issue is how to study the effective substance of ZQ to play a key role in depression. Huang xi suggested a tactics of bioethnopharmaceutical analytical pharmacology (BAP). In brief, BAP is a conceptual framework for illuminating the absorbed bioactive compounds (ABCs) in TCM formulas or herbal remedies (Wang et al., 2011). In general, ABCs are more possibly to play a part in the therapeutic effect *in vivo* after oral administration (Zhang et al., 2017b). Thus, it is necessary to measure the ABCs in the target organ to understand the effective substances in the herb. Additionally, the occurrence of depression is closely related with the hippocampus and cortex (Chen et al., 2017). Therefore, in the present study, the components of ZQ were qualitatively analyzed in plasma, the hippocampus and the cortex after oral administration in acute depressive rats.

At present, some studies on ZQ have been conducted, including studies of the chemical constituents, pharmacology, pharmacokinetics and clinical remedy. According to literature, 5 constituents of the aqueous extract of ZQ were detected by UPLC-PDA (Xing et al., 2014). Thirty-one components in the methanol extract of ZQ were identified using LC/MS-MS (Zhang et al., 2014). As a main constituent of ZQ, the pharmacokinetics of meranzin hydrate (MH) in rat was recently reported (Xie et al., 2013). Meanwhile, the pharmacokinetics of hesperidin, naringin, neohesperidin, naringenin and hesperetin were studied in healthy rats after receiving oral ZQ extract (Tong et al., 2012). However, the pharmacokinetics of the 7 compounds in ZQ following oral administration to depressive rats remains unstudied. In our study, a rapid and selective LC/MS/MS method was developed for the simultaneous determination of the 7 bioactive components. Additionally, pharmacokinetic compounds were closely linked to the antidepressant effect of ZQ in rats.

## Materials and Methods

### Materials

The reference standards for naringenin, nobiletin, narirutin, naringin, hesperidin, neohesperidin, sulfamethoxazole (internal standard, IS1), and honokiol (internal standard, IS2) were purchased from the Shanghai Yuanye Bio-Technology Co., Ltd.(Shanghai, China). Meranzin hydrate (MH) was obtained from the Huaxi Medical University Medicine Factory (Chengdu, China). Formic acid, methanol and acetonitrile (HPLC grade) were purchased from Sigma (United States). Water was purified by a Milli-Q water purification system (Millipore, Burlington, MA, United States). ZQ was obtained from the pharmacy in Xiangya Hospital, Central South University and authenticated by Professor S. Y. Hu.

### Apparatus and Operating Conditions

Chromatographic analysis was carried out on an Agilent Technologies 1200 series LC system (Agilent Corporation, Santa Clara, CA, United States), equipped with a G1311A quaternary pump, a G1329A autosampler, a G1322A on-line degasser, and a G1316A thermostat. The chromatographic separation was achieved on an 300SB-C18 column (4.6 mm × 250 mm, i.d., 5 μm, Agilent, United States) and an equivalent guard column (5 μm, Phenomenex, United States). Gradient elution with 0.1% formic acid (A) and acetonitrile (B) was applied for chromatographic separation at a flow rate of 1.0 mL/min. The mobile phase was used as follows: 23% B from 0 to 6.0 min; 23–65% B from 6.0 to 6.5 min; 65–65% B from 6.5 to 11.5 min; 65–23% B from 11.5–12.0 min; 23% B from 12.0 to 18.0 min. The total analysis time was 18.0 min, and the injection volume was 10 μL.

An ABI 3200Q-TRAP quadrupole linear ion trap mass spectrometer with an electrospray ion source (ESI) was used in this study. The ESI source was operated in the positive ionization mode for naringenin, nobiletin, MH and sulfamethoxazole and in the negative ion mode for narirutin, naringin, hesperidin, neohesperidin and honokiol. The precursor-to-product ion transitions in multiple reaction monitoring mode (MRM) were used for mass analysis and quantification. The precursor-to-production pairs and optimized declustering potential, entrance potential, collision energy and collision cell exit potential for each analyte and IS are showed in **Table 1**. Other MS/MS setting conditions were as follows: curtain gas pressure at 20 psi, ion source gas 1 at 60 psi, ion source gas 2 at 55 psi, ion spray voltage at 5500 V (in positive ion source) or -4500 V (in negative ion source), and temperature at 600°C. High purity nitrogen was employed as both the nebulizing and drying gasses. The process was performed by Analyst 1.4.1 data acquisition and processing software (applied Biosystems/MDS Sciex).

**Table 1:** Retention (t_R_), MRM transitions in positive or negative ion mode and parameters for the determination of 7 compounds and IS.

Analyte	*t*_R_ (min)	Transition monitored (Q1/Q3)	DP	EP	CEP	CE	CXP
Naringenin	10.35	273.2/152.9	42.5	10.0	17.63	30.0	3.0
Nobiletin	11.24	403.0/373.0	107.5	6.0	21.26	33.0	5.0
Meranzin hydrate	7.65	279.3/189.1	48.0	6.1	17.80	21.0	4.0
Narirutin	4.68	579.0/271.2	-150.0	-6.0	-32.94	-45.0	-2.5
Naringin	5.13	579.0/271.2	-260.0	-7.0	-20.0	-35.0	-2.0
Hesperidin	5.43	609.0/300.4	-55.0	-6.5	-43.5	-33.0	-3.0
Neohesperidin	6.02	609.0/300.4	-80.0	-7.0	-40.0	-36.0	-3.0
Honokiol	12.08	265.4/222.8	-65.0	-10.0	-17.3	-44.5	-4.0
Sulfamethoxazole	6.92	254.1/107.7	50.0	10.0	17.09	30.0	5.0


### Preparation of ZQ Extract

The crude ZQ was extracted according to our previously described method (Zhang et al., 2017a). Briefly, the ZQ was infiltrated in distilled water (1:12, w/v) for 30 min and boiled twice for 30 min under 100°C. The two decoctions were merged, evaporated under pressure at 60°C, and lyophilized, outputting the ZQ lyophilized powder with a yield of 19.7%. To count the dosage, the contents of the main compounds in the ZQ extract were quantitatively measured using the same analysis conditions as described previously. In additionally, the lyophilized powder was evenly dispersed in distilled water at dosage of ZQ (20 g/kg) before the experiment.

### Animal Experiments and Drug Administration

All animal experimental procedures were performed in line with the guidelines for the institutional guidelines of the Animal Care and Use Committee of Central South University (Changsha, China). The protocol was approved by the animal experimental committee of Central South University. All efforts were used to minimize the pain and suffering of the rats. SPF male Sprague–Dawley rats (220–250 g) were supplied by Shanghai SLAC Laboratory Animal Co. Ltd. (Shanghai, China), and housed under ambient temperature of 23 ± 2°C with a 12 h light/dark cycle and 50% relative humidity in the laboratory, and free access to a normal standard chow diet and water. All the rats were fasted for 12 h with free access to tap water prior to the experiment.

ZQ lyophilized powder or fluoxetine (Eli Lilly, United States) was dissolved with saline. To evaluate dose-dependent effects, ZQ (5, 10, 20 g/kg) or fluoxetine (20 mg/kg) was orally administered as a single dosage 0.5 h before the test. To study the precise relationship between antidepressant effects and pharmacokinetics, ZQ (20 g/kg) was orally administered as a single dose 0.5, 1, 2, or 4 h before the test. Similarly, vehicle rats were orally administered an equal volume of saline using the identical procedure.

### Forced Swimming Test (FST)

The swimming tests were conducted according to a previously described method (Flores-Burgess et al., 2017) with minor modifications. In brief, all experiments were operated between 12:00 h and 15:00 h in the light cycle. Each rat was individually put into a vertical glass cylinder with a diameter of 20 cm, water depth of 30 cm and water temperature of 25°C. A 15-min forced swimming test was conducted 24 h before the 5-min test. Each rat was deemed to be immobile when it stopped scrabbling, remained floating stilly in the water and only made necessary struggling to maintain its head over water. The total duration of immobility was recorded during the 5-min test.

### Preparation of Stock and Working Solutions, Calibration Standards, and Quality Control Samples

Standard stock solutions of naringenin (2420 ng/mL), nobiletin (1750 ng/mL), MH (10480 ng/mL), narirutin (5720 ng/mL), naringin (5190 ng/mL), and hesperidin (2375 ng/mL) were prepared in methanol, and neohesperidin (3640 ng/mL) was dissolved in hot water. Then, the calibration standard stock solutions of gradient concentrations were obtained by serial dilution with 90% methanol/water. The internal standard (IS) solutions of sulfamethoxazole and honokiol were prepared to the concentrations of 754 ng/mL and 274 ng/mL in methanol, respectively. All working solutions were enclosed and stockpiled at 4°C, and then they were returned to room temperature before test.

The calibration standards were prepared by spiking 20 μL of appropriate standard working solution into 180 μL of blank plasma to obtain the concentrations ranging from 242.0 to 3.78 ng/mL for naringenin, 175.0–2.73 ng/mL for nobiletin, 1048.0–16.38 ng/mL for MH, 572.0–8.94 ng/mL for narirutin, 519.0–8.11 ng/mL for naringin, 237.5–3.71 ng/mL for hesperidin, and 364.0–5.69 ng/mL for neohesperidin.

Quality control (QC) samples at low, middle and high concentrations (3.78, 30.25, and 242.0 ng/mL for naringenin; 2.73, 21.88, and 175.0 ng/mL for nobiletin; 16.38, 131.0, and 1048.0 ng/mL for MH; 8.94, 71.5, and 572.0 ng/mL for narirutin; 8.11, 64.88, and 519.0 ng/mL for naringin; 3.71, 29.69, and 237.5 ng/mL for hesperidin; and 5.69, 45.5, and 364.0 ng/mL for neohesperidin) were also prepared by the same procedures. The QC samples in plasma were extracted in the same way as the standards samples on each analysis day.

### Biological Sample Preparation

To identify the main active compounds of ZQ in target tissue, six rats subjected to FST were sacrificed under anesthesia 0.5 h after oral administration of ZQ extract (20 g/kg), and then the hippocampal and cortex tissue was separated rapidly from the brain on an ice tray and stored at -80°C. All tissues (∼50.0 mg) were homogenized with 1.5 mL of ice-cold 0.9% saline.

Sample (200 μL) and 10 μL of mixed IS solution containing sulfamethoxazole (754 ng/mL) and honokiol (274 ng/mL) was added to Eppendorf tubes and extracted with 1 mL acetonitrile. The mixed solution was vortexed for 3 min and centrifuged at 10000 rpm for 10 min at 4°C to acquire the supernatant. The supernatant (800 μL) was moved to a clean Eppendorf tube, and then desiccated under a flow of nitrogen at 40°C. Finally, the residue was redissolved in 100 μL acetonitrile/water (90:10, v/v) with vortex-mixing for 3 min and centrifuged at 13000 rpm for 10 min. A 10 μL aliquot of the sample was injected into the LC/MS/MS system for analysis.

### Method Validation

The analytical method was validated for its selectivity, linearity, sensitivity, accuracy, precision, matrix effect, recovery and stability according to US FDA bioanalytical method validation guidance (Zhang et al., 2017b).

### Specificity and Selectivity

Blank rat plasma samples or samples spiked with the IS were detected for endogenous or IS interference. Specificity and selectivity were investigated by comparing the chromatograms of six individual batches of blank rat plasma samples, plasma samples added with the 7 analytes and IS, and plasma samples 30 min after oral administration of ZQ extracts.

### Linearity

The calibration curve was obtained by plotting the peak area ratio (y) of the analytes to IS vs. the plasma concentration (x) of the analytes with weighted (1/x) least square linear regression. The lower limits of quantification (LLOQ) was conformed to the lowest concentration on the standard curve with an acceptable accuracy (relative error, RE ≤± 20%) and a precision (relative standard deviation, RSD ≤ 20%).

### Precision and Accuracy

The intra- and inter-day precisions were assessed by RSD, and developed by analyzing three concentrations levels of QC samples. Six samples were measured for each concentration level on the same experimental day and the three successive days, respectively. The analytic precision was denoted as the RSD, and the accuracy was considered the RE of the measured average deviated from the nominal value. The acceptable norm for the RSD was required to be less than 15%, and the RE was required to be within ±15%.

### Recovery and Matrix Effect

The extraction recoveries of the analytes were calculated by comparing the peak areas gained from six plasma samples in the pre-extraction spiked with the analytes and the post-extraction at three QC levels. The matrix effects were evaluated by contrast the peak areas obtained from the plasma matrix added with working standard solutions with the pure standard solutions at the same concentrations. The extraction recovery and matrix effect of the IS were also measured by the same approach at identical concentration.

### Stability Experiment

The stabilities of the 7 analytes in rat plasma was evaluated by assaying six replicates of QC samples at low, middle, and high concentrations in practical experimental conditions: samples post-preparation stored in auto-sampler condition for 12 h, and storage at -80°C for 1 month. The freeze- thaw stability was determined after the experience of three freeze-thaw cycles (from -80°C to room temperature). Samples were identified as stable if the accuracy bias was within 15.0% of the nominal concentration.

### Pharmacokinetic Study

All of the rats were subjected to FST for 24 h before oral administration of the ZQ extract (20 g/kg). Plasma samples (about 0.5 mL) were gathered in a heparinized 1.5 mL Eppendorf tube from the venous plexus of the eye socket at 0.083, 0.25, 0.5, 0.75, 1.0, 1.5, 2.0, 3.0, 4.0, 6.0, 8.0, 12.0, and 24.0 h under anesthesia. Each sample was immediately centrifuged at 10000 rpm for 15 min to acquire the plasma. At last, the plasma was immediately placed at -80°C until measure.

### Data Analysis

All data were expressed as the mean ± SD. The plasma concentration of the 7 compounds during the experiment was calculated according to the daily calibration curve. The pharmacokinetic parameters, including C_max_ (the peak drug plasma concentration), T_max_ (the time to C_max_), AUC_0-t_ (the area under the plasma concentration-time curve from 0 to time), AUC_0-∞_ (the area under the plasma concentration-time curve from 0 to time infinity), and *t*_1/2_ (the elimination half-life) were calculated by performing a non-compartment model using WinNonlin Professional Edition Version 2.1 (Pharsight Corporation, Sunnyvale, CA, United States). The immobile time in the FST test was evaluated by one-way ANOVA. Statistical significance was denoted at *p* < 0.05. Statistical analysis was operated by GraphPad Prism 5 software.

## Results and Discussion

### Contents of Main Active Compounds in ZQ Extract

The analytical results indicated that the contents of naringenin, nobiletin, Meranzin hydrate (MH), narirutin, naringin, hesperidin and neohesperidin in ZQ extract were 0.075, 0.067, 0.011, 0.220, 1.233, 0.122, and 1.471 mg/g, respectively. Quantitative data also showed that naringin and neohesperidin were the more predominant flavonoids in ZQ extract. Furthermore, it is reported that the contents of the ingredients in ZQ were significantly affected by different geographical origins (Li et al., 2016).

### Effects of ZQ on Immobility Time During Forced Swimming Test (FST) After Acute Treatment

**FIGURE 1:**
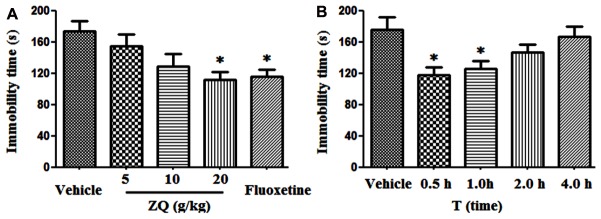
Effects of acute ZQ treatment on immobility time in rats undergoing the forced swimming test (FST). ZQ (5, 10, or 20 g/kg) and Fluoxetine (20 mg/kg) were orally administered 0.5 h prior to the behavioral test **(A)**. Effect of an acute oral administration of ZQ (20 g/kg) 0.5, 1, 2, or 4 h prior to the behavioral test **(B)**. Data are represented as the mean ± SEM, *n* = 7–8, ^∗^*p* < 0.05 versus the vehicle group.

ZQ (20 g/kg) and Fluoxetine (20 mg/kg) significantly decreased the immobility time of rats (111.39 ± 33.26, 116.07 ± 28.65 s) compared with the vehicle (173.18 ± 40.36 s, *p* < 0.05) in the FST (**Figure 1**). However, there were no statistically significant discrepancy in the ZQ extract (5 or 10 g/kg) (128.89 ± 44.71, 154.43 ± 43.94 s, *p* > 0.05). In line with previous studies (Zhang Y.J. et al., 2012), our results further confirmed the antidepressant effect of ZQ against FST by decreasing immobility time. The FST is used as a valid behavioral test to assess the efficacy of antidepressant drugs (Flores-Burgess et al., 2017). That is, the FST is also applied to survey the behavioral despair as well as passive, coping and negative behaviors which are regarded as a main symptoms of depression (Slattery and Cryan, 2012; Haj-Mirzaian et al., 2016). The behavioral despair is an act of “giving up hope of escaping” after stress, which can be reflected by the immobile behavior in animal modes of depression (Cryan et al., 2005). As such, remarkable reduction in immobility time in this test after giving drug is considered as a main indication of an antidepressant-like action.

In additional, the relationship between the antidepressant effect and pharmacokinetics was precisely investigated. As shown in **Figure 1B**, the immobility time was significantly reduced following oral administration of ZQ (20 g/kg) at 0.5 and 1 h (117.79 ± 26.55, 125.27 ± 27.54 s) before the test compared with the vehicle (175.24 ± 42.57 s, *p* < 0.05), whereas no significant decrease was observed at 2 and 4 h (146.53 ± 26.39, 166.81 ± 34.51 s) before the test. Many phytochemicals have been revealed in ZQ extract (Duan et al., 2014), however, absorbed bioactive compounds (ABCs) in ZQ *in vivo* remains unknown.

### Optimization of the Analytic Conditions

We optimized the mobile phase to obtain good chromatographic conditions. Compared to methanol, the mass response and resolution of the analytes and IS were greatly enhanced by acetonitrile instead of methanol after several trial tests. Additionally, peak symmetry and sensitivity of naringenin, narirutin and hesperidin were significantly perfected by the addition of 0.1% formic acid. Isomers of narirutin vs. naringin and hesperidin vs. neohesperidin have almost close polarity and identical precursor-to-product ion pairs in MS. Therefore, two pairs of isomers were separated completely with different retention times by optimization of the gradient elution (**Figure 3**). Similarly, the run time was also shortened in our study.

**FIGURE 2:**
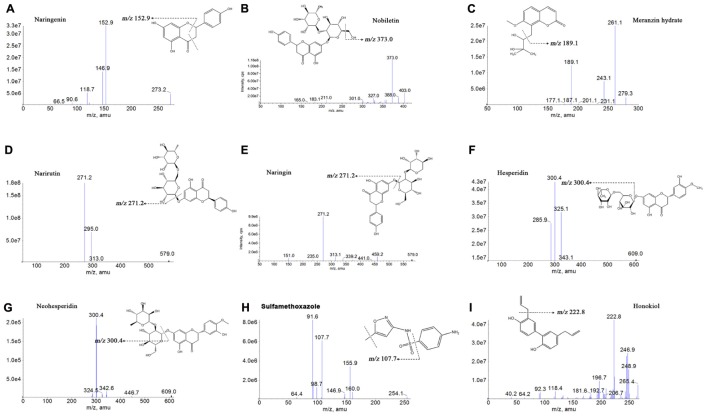
Chemical structures and product ion mass spectra of naringenin **(A)**, nobiletin **(B)**, meranzin hydrate **(C)**, narirutin **(D)**, naringin **(E)**, hesperidin **(F)**, neohesperidin **(G)**, sulfamethoxazole **(H)**, and honokiol **(I)**.

The standard solutions of the 7 analytes and IS were directly injected along with the mobile phase into the mass spectrometer with an ESI source. To seek more strong response values of all analytes and IS, both the positive and negative ionization modes were compared. The response of naringenin, nobiletin, MH and sulfamethoxazole surveyed in the positive [M-H]^+^ ionization mode was stronger compared with the negative ionization mode in the experiment. However, the response in the negative [M-H]^-^ ionization mode became higher than that in the positive ionization mode for narirutin, naringin, hesperidin, neohesperidin and honokiol. **Figure 2** explains the product ion scan spectra of the analytes and IS. The MS/MS transitions and energy parameters of the 7 compounds and IS are listed in **Table 1**.

### Optimization of the Sample Preparation

Due to their different pKa values, dissolution properties and concentrations as well as stabilities in the serous matrix, the sample extraction method for the various analytes in plasma was optimized, and the extraction procedure should be simple, time-saving, repeatable, minimize matrix effects and be as convenient as possible. To achieve desired recovery of the multiple compounds, liquid-liquid extraction (LLE) was applied because this method could concentrate the analytes. Therefore, acetonitrile, instead of methanol, was eventually chosen to obtain higher extraction recovery in our experiment.

### Chromatographic Analysis of Active Compounds in ZQ Extracts

**FIGURE 3:**
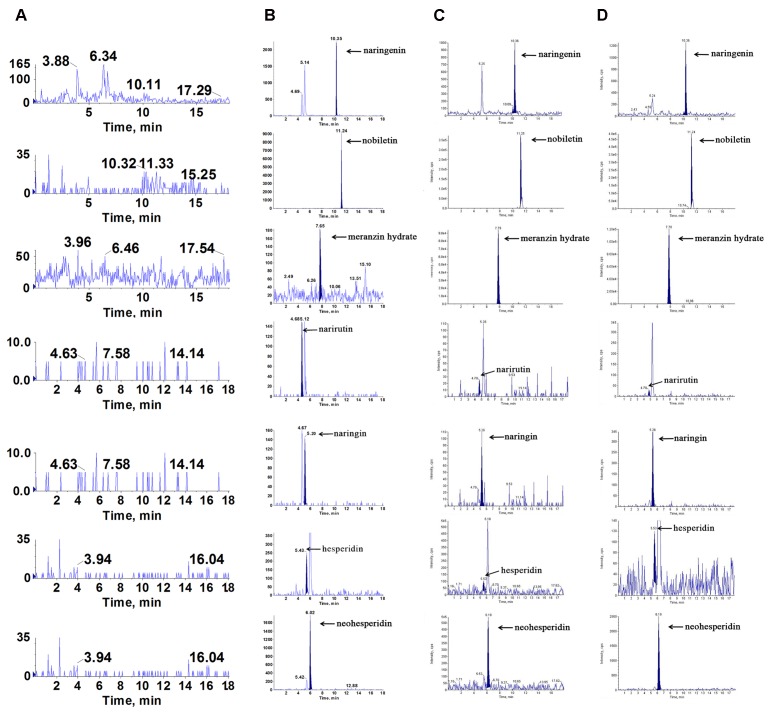
Representative multiple reaction monitoring (MRM) chromatograms of naringenin, nobiletin, meranzin hydrate, narirutin, naringin, hesperidin and neohesperidin *in vivo*: **(A)** blank plasma, **(B)** blank plasma spiked with the 7 analytes, **(C)** hippocampus and **(D)** cortex were obtained at 0.5 h after a single oral administration of ZQ extract (20 g/kg) to depressed rats.

To find what active components were absorbed in the blood and brain, which played latently acute antidepressant effect, we qualitatively assayed the samples by LC/MS-MS. Compared with the retention of the standards, we found that naringenin, nobiletin, MH, narirutin, naringin, hesperidin and neohesperidin were absorbed into the hippocampus and cortex of acute depressive rats after oral administration of ZQ extract (**Figure 3**). Interestingly, it has been reported that naringenin, nobiletin, MH, naringin and hesperidin play a protective role against acute depression (Pathak et al., 2013; Yi et al., 2014; Choi et al., 2017). Therefore, these findings support the possibility that these are the main active compounds of ZQ, which are representative of its antidepressant effect. However, the precise relationship between the antidepressant effects of ZQ and the pharmacokinetics remains unknown. This issue is a challenge to understanding pharmacokinetic significance and the use of ZQ.

### Method Validation

#### Selectivity

**FIGURE 4:**
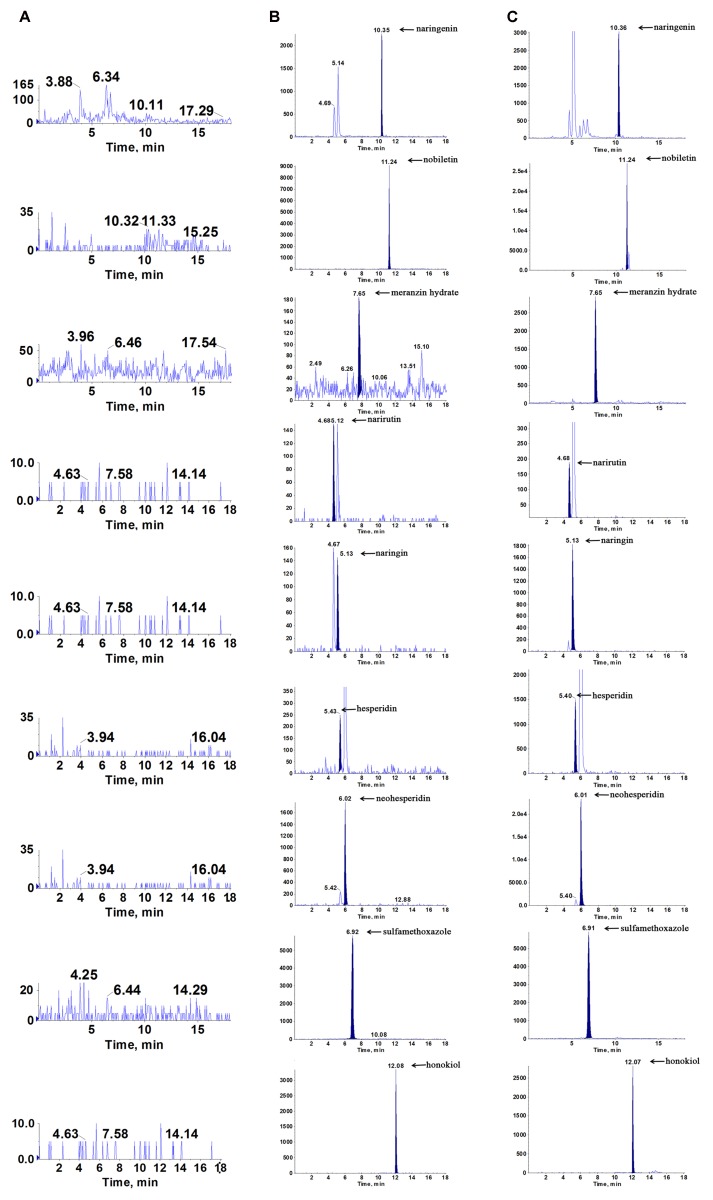
Representative multiple reaction monitoring (MRM) chromatograms of naringenin, nobiletin, meranzin hydrate, narirutin, naringin, hesperidin, neohesperidin, sulfamethoxazole (IS1) and honokiol (IS) in rat plasma: **(A)** blank plasma, **(B)** blank plasma spiked with the 7 analytes and IS at LLOQ, and **(C)** plasma sample collected at 0.5 h after a single oral administration of ZQ extract (20 g/kg) to depressive rats.

The representative chromatograms acquired from blank plasma, blank plasma added with the 7 analytes at lower limits of quantification (LLOQ) and IS, and plasma sample following oral administration of the ZQ extract are shown in **Figure 4**. Due to the efficient sample preparation and high selectivity of MRM, there was no obvious endogenous interference in the plasma and the 7 compounds and IS could be separated completely from each other.

#### Linearity and Sensitivity

As shown in **Table 2**, the equations and correlation coefficients (*r*) were as follows: *y* = 0.0354x + 0.0050, *r* = 0.9970 for naringenin, *y* = 0.0476x +0.1877, *r* = 0.9991 for nobiletin, *y* = 0.0026x – 0.0601, *r* = 0.9987 for MH, *y* = 0.0081x + 0.0167, *r* = 0.9999 for narirutin, *y* = 0.0078x + 0.0343, *r* = 0.9996 for naringin, *y* = 0.0199x + 0.0379, *r* = 0.9998 for hesperidin, and *y* = 0.1342x + 0.3445, *r* = 0.9999 for neohesperidin in the plasma. The LLOQ of naringenin, nobiletin, MH, narirutin, naringin, hesperidin and neohesperidin were 3.78, 2.73, 16.38, 8.94, 8.11, 3.71, and 5.69 ng/mL, respectively.

**Table 2:** The regression equations, linear ranges and LLOQs of 7 compounds in rat plasma.

Analyte	Calibration curves	Range (ng/mL)	Correlation coefficient (*r*)	LLOQ (ng/mL)
Naringenin	*y* = 0.0316x + 0.1260	3.78 – 242.0	0.9994	3.78
Nobiletin	*y* = 0.047x + 0.1810	2.73 – 175.0	0.9995	2.73
Meranzin hydrate	*y* = 0.0026x - 0.0601	16.38 – 1048.0	0.9987	16.38
Narirutin	*y* = 0.0081x + 0.0167	8.94 – 572.0	0.9999	8.94
Naringin	*y* = 0.0078x + 0.0343	8.11 – 519.0	0.9996	8.11
Hesperidin	*y* = 0.0199x + 0.0379	3.71 – 237.5	0.9998	3.71
Neohesperidin	*y* = 0.1342x + 0.3445	5.69 – 364.0	0.9999	5.69


#### Precision and Accuracy

As shown in **Table 3**, the data illustrated that the method had good precision and accuracy. For the intra-day precisions varied from 2.78 to 11.00%, while the accuracy ranged from -10.57 to 10.68% for each quality control (QC) level of the analytes. Similarly, the inter-day precisions ranged from 4.17% to 13.17%. While, the accuracy between -10.19 and 9.13%. The precision and accuracy were all within the acceptable range for working in biological media.

**Table 3:** Intra- and inter-day precisions and accuracies of the 7 analytes from QC samples prepared in rat plasma (*n* = 6).

Analytes	Nominal conc (ng/mL)	Intra-day			Intre-day		
			
		Observed	Precision (RSD, %)	Accuracy (RE, %)	Observed	Precision (RSD, %)	Accuracy (RE, %)
Naringenin	3.8	3.38 ± 0.30	8.94	10.57	3.98 ± 0.34	8.49	0.05
	30.3	28.28 ± 2.74	9.70	-6.50	32.16 ± 4.24	13.17	6.31
	242.0	238.91 ± 17.89	7.49	-1.28	244.60 ± 10.21	4.17	1.08
Nobiletin	2.7	2.53 ± 0.23	9.03	-7.58	2.45 ± 0.24	9.94	-10.19
	21.9	20.78 ± 1.86	8.97	-5.02	21.39 ± 1.97	9.22	-2.21
	175.0	174.20 ± 13.67	7.84	-0.46	171.87 ± 9.44	5.49	-1.79
Meranzin hydrate	16.4	17.35 ± 0.98	2.81	5.96	17.87 ± 3.03	8.46	9.13
	131.0	123.33 ± 6.20	5.03	-5.85	128.34 ± 9.35	7.30	-2.03
	1048.0	1036.8 ± 55.74	5.38	-1.07	1109.02 ± 91.15	8.22	5.82
Narirutin	8.9	9.55 ± 0.59	6.17	6.77	9.09 ± 0.62	6.86	1.75
	71.5	77.53 ± 5.82	7.50	8.44	75.13 ± 4.02	5.35	5.08
	572.0	616.52 ± 43.05	6.98	7.78	586.90 ± 49.60	8.45	2.61
Naringin	8.1	8.98 ± 0.73	8.16	10.68	8.28 ± 0.79	9.50	2.15
	64.9	70.18 ± 7.72	11.00	8.17	68.68 ± 4.49	6.55	5.85
	519.0	524.76 ± 34.41	6.56	1.11	550.19 ± 29.78	5.41	6.01
Hesperidin	3.7	3.85 ± 0.11	2.78	3.66	3.98 ± 0.29	7.30	7.28
	29.7	32.13 ± 1.32	4.12	8.23	31.10 ± 1.58	5.08	4.75
	237.5	253.52 ± 10.61	4.19	6.75	251.14 ± 19.63	7.82	5.74
Neohesperidin	5.7	6.29 ± 0.41	6.51	10.59	5.24 ± 0.60	11.54	-7.92
	45.5	48.22 ± 1.72	3.56	5.98	49.42 ± 2.83	5.73	8.62
	364.0	385.44 ± 24.49	6.35	5.89	371.43 ± 21.99	5.92	2.04


#### Extraction Recovery and Matrix Effects

The mean extraction recovery and matrix effects of three level QC samples were listed in **Table 4**. The recovery of sulfamethoxazole (IS1) and honokiol (IS2) were 82.17 ± 14.41% and 77.41 ± 8.07%, respectively. The matrix effects for the 7 analytes and IS were between 81.53 and 118.52%. Besides, all of the RSD values were under 15.00%. Obviously, co-eluting endogenous substances would not markedly disturb the analysis of the rat plasma.

**Table 4:** Recovery and matrix effects of the 7 analytes in rat plasma (*n* = 6).

Analyte	Nominal conc (ng/mL)	Recovery		Matrix effect	
			
		Mean ± *SD* (%)	RSD (%)	Mean ± *SD* (%)	RSD (%)
Naringenin	3.8	78.41 ± 7.08	9.03	96.03 ± 2.19	2.28
	30.3	82.39 ± 7.78	9.44	85.77 ± 3.29	3.84
	242.0	91.79 ± 2.98	3.25	79.38 ± 3.31	4.17
Nobiletin	2.7	77.81 ± 6.31	8.11	81.53 ± 8.09	9.92
	21.9	80.06 ± 5.14	6.42	82.05 ± 6.61	8.06
	175.0	85.09 ± 6.06	7.12	86.41 ± 5.10	5.90
Meranzin hydrate	16.4	82.38 ± 8.07	9.79	110.79 ± 3.64	3.29
	131.0	106.44 ± 5.13	4.82	103.86 ± 4.56	4.39
	1048.0	89.26 ± 1.95	2.19	101.22 ± 1.35	1.33
Narirutin	8.9	79.56 ± 6.72	8.45	100.33 ± 7.41	7.38
	71.5	92.40 ± 3.92	4.24	99.15 ± 9.25	9.33
	572.0	98.32 ± 7.56	7.69	99.93 ± 5.67	5.68
Naringin	8.1	78.22 ± 4.85	6.21	99.04 ± 9.04	9.13
	64.9	91.31 ± 5.12	5.61	98.63 ± 4.19	4.25
	519.0	101.12 ± 3.62	3.58	102.70 ± 2.09	2.03
Hesperidin	3.7	79.31 ± 4.94	6.23	118.52 ± 10.23	8.63
	29.7	83.33 ± 3.04	3.65	94.88 ± 5.15	5.43
	237.5	94.30 ± 3.48	3.69	99.73 ± 2.87	2.88
Neohesperidin	5.7	79.91 ± 4.25	5.32	109.99 ± 5.86	5.33
	45.5	86.88 ± 1.86	2.14	98.57 ± 1.42	1.49
	364.0	98.64 ± 3.29	3.33	99.51 ± 1.89	1.91


#### Stability

The stabilities of the 7 analytes were fully evaluated by QC sample analysis during the sample storing and processing procedures.

The results (**Table 5**) showed that all compounds were stable in plasma for 12 h in auto-sampler condition after preparation, three freeze-thaw cycles and 1 month storage at -80°C, with all RE values in the range from -13.99 to 12.29%.

**Table 5:** Stability of the 7 analytes in rat plasma (*n* = 6).

Analyte	Nominal Cone (ng/mL)	Room temperature for 12 h			Three freeze-thaw cycles			-80°C for 1 month		
				
			RSD (%)	RE (%)		RSD (%)	RE (%)		RSD (%)	RE (%)
Naringenin	3.8	3.99 ± 0.30	7.63	5.64	3.44 ± 0.36	10.59	-8.95	3.50 ± 0.38	10.98	-7.39
	30.3	33.48 ± 2.30	6.86	10.67	28.90 ± 2.52	8.71	-4.45	28.43 ± 3.57	12.55	-6.00
	242.0	235.80 ± 6.33	2.69	-2.56	235.73 ± 13.62	5.78	-2.59	249.60 ± 19.69	7.89	3.14
Nobiletin	2.7	2.61 ± 0.23	8.95	-4.37	2.52 ± 0.28	11.31	-7.88	2.46 ± 0.31	12.70	-9.94
	21.9	22.97 ± 2.46	10.72	5.03	20.21 ± 2.65	13.13	-7.62	20.09 ± 1.33	6.64	-8.19
	175.0	182.84 ± 8.80	4.81	4.48	163.22 ± 5.69	3.48	-6.73	173.01 ± 12.94	7.48	-1.14
Meranzin hydrate	16.4	18.22 ± 1.86	5.12	11.26	18.33 ± 2.58	7.03	11.95	17.52 ± 1.49	4.26	6.97
	131.0	134.48 ± 10.24	7.62	2.66	112.67 ± 8.81	7.82	-13.99	140.55 ± 1.39	0.996	7.29
	1048.0	1124.97 ± 93.69	8.33	7.34	1084.48 ± 115.67	10.67	3.48	1146.65 ± 74.29	6.48	9.41
Nariratin	8.9	10.05 ± 1.48	14.72	12.43	9.80 ± 1.07	10.90	9.69	9.69 ± 0.85	8.74	8.49
	71.5	72.82 ± 2.13	2.92	1.84	77.85 ± 2.47	3.17	8.89	76.91 ± 8.39	10.91	7.57
	572.0	601.89 ± 36.15	6.01	5.53	617.42 ± 56.25	9.11	7.94	589.30 ± 42.03	7.13	3.03
Naringin	8.1	8.58 ± 0.86	9.99	5.84	8.87 ± 1.03	11.67	9.33	9.11 ± 0.66	7.29	12.29
	64.9	68.37 ± 2.90	4.24	6.83	67.69 ± 4.02	5.94	4.34	65.02 ± 5.42	8.33	0.21
	519.0	549.85 ± 45.89	8.35	5.94	521.88 ± 23.35	4.47	0.56	542.71 ± 54.54	10.05	4.57
Hesperidin	3.7	3.64 ± 0.36	9.99	-1.88	3.86 ± 0.45	11.65	4.08	3.95 ± 0.30	7.70	6.36
	29.7	29.96 ± 1.22	4.06	0.91	31.41 ± 1.87	5.97	5.80	30.92 ± 2.13	6.88	4.15
	237.5	249.08 ± 7.29	2.93	4.88	233.64 ± 11.32	4.85	-1.62	237.54 ± 13.73	5.78	0.02
Neohesperidin	5.7	5.36 ± 0.58	10.87	-5.80	6.10 049	8.04	7.23	5.88 ± 0.59	10.11	3.31
	45.5	48.81 ± 3.71	7.61	7.28	48.18 ± 4.65	9.64	5.88	46.92 ± 4.29	9.16	3.13
	364.0	403.38 ± 24.69	6.12	10.82	362.91 ± 19.95	5.49	-0.30	371.39 ± 25.53	6.87	2.03


#### Pharmacokinetic Study

**FIGURE 5:**
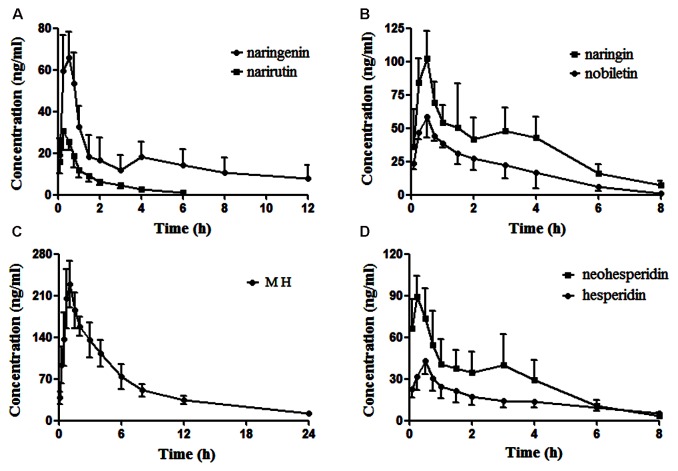
The profiles of the mean plasma concentration over time for naringenin and narirutin **(A)**, naringin and nobiletin **(B)**, meranzin hydrate (MH) **(C)**, and neohesperidin and hesperidin **(D)** after oral administration of ZQ extract (20 g/kg) to rats (mean ± SD, *n* = 6).

Naringenin, nobiletin, MH, narirutin, naringin, hesperidin and neohesperidin were successfully identified in the target tissue of rats subjected to acute depression. It seemed to confirm that these compounds are the effective substances of ZQ that possess an antidepressant effect. Here, the pharmacokinetic compounds were precisely linked with the antidepressant effects of ZQ. The developed assay was sensitive enough to applied in the pharmacokinetic study of 7 components in rat plasma following oral administration of ZQ extract at a dose of 20 g/kg. The plasma concentration-time curve of the 7 analytes in rat plasma are presented in **Figure 5**, and the obtained pharmacokinetic parameters are summarized in **Table 6**.

**Table 6:** Pharmacokinetic parameters of the 7 investigated compounds in male SD rats after oral administration of extract ZQ (mean ± SD, *n* = 6).

	T_max_ (h)	C_max_ (ng/L)	*T*_1/2_ (h)	AUC_0-t_ (ng h/L)	AUC_o-oo_ (μg h/L)
Naringenin	0.42 ± 0.13	78.08 ± 22.23	4.37 ± 1.19	187.55 ± 38.86	225.68 ± 64.93
Nobiletin	0.38 ± 0.14	53.88 ± 14.29	1.28 ± 0.87	150.59 ± 40.59	153.40 ± 39.99
Meranzin hydrate	1.25 ± 0.42	221.99 ± 53.94	7.31 ± 1.51	1475.97 ± 259.46	1706.42 ± 350.46
Narirutin	0.33 ± 0.13	31.14 ± 8.83	1.49 ± 0.42	42.61 ± 9.64	44.85 ± 9.99
Naringin	0.42 ± 0.13	103.75 ± 18.78	1.67 ± 0.57	292.56 ± 91.97	311.94 ± 89.49
Hesperidin	0.42 ± 0.13	45.96 ± 7.32	2.93 ± 0.88	136.31 ± 23.69	179.42 ± 43.87
Neohesperidin	0.29 ± 0.10	90.76 ± 48.55	2.02 ± 0.77	234.09 ± 87.33	238.73 ± 95.59


As shown in **Figure 5**, in the plasma concentration-time profiles of naringin, naringenin and neohesperidin, a small peak is appeared that was consistent with the literature (Tong et al., 2012; Zhang J. et al., 2012). The AUC_0-t_ of naringenin and nobiletin are close to those of the glycosides (narirutin, naringin, hesperidin, and neohesperidin), whereas the content of the glycosides are rather higher than that of naringenin. This phenomenon may be due to the hydrolysis of the glycosides mediated by gastrointestinal bacteria via oral administration (Hsiu et al., 2002; Tong et al., 2012). As a metabolite of naringin *in vivo*, the AUC_0-∞_ of naringenin is slightly lower than naringin, although the administered doses of naringenin was approximately 16-fold less than that of naringin in ZQ extract. According to an early paper (Tong et al., 2012), the AUC_0-∞_ of naringenin, naringin and neohesperidin in healthy rats was higher than the data obtained in our experiment after oral administration of ZQ at dose of 3.2 g/kg. However, the dosage of administration in our study is far greater than them. Furthermore, depression is often accompanied by functional dyspepsia (Adibi et al., 2016), resulting in changes in gastrointestinal bacteria and absorptive capacity. This change explains that the lower AUC_0-∞_ of the main components in depressive rats following oral administration of ZQ extract (20 g/kg) compared with healthy rats. However, above precise linkage needs to further establish. Surprisingly, the content of MH was the lowest in the 7 compounds of ZQ. However, the AUC_0-∞_ of MH was at least fivefold higher than other flavonoids, and its T_1/2_ exceeded 7.3 h, probably due to its different chemical structure and physicochemical properties. Additionally, the 7 components were rapidly absorbed *in vivo*, resulting in the highest concentration within 0.5 h following the oral administration of ZQ extract. As shown in **Figure 2B**, the antidepressant activity was increased significantly at 0.5 or 1 h following oral administration of ZQ before the test compared with 2 or 4 h after administration. These consequences might offer a guidance to further pharmacodynamics researches of ZQ.

## Conclusion

An rapid and sensitive LC-MS/MS method was first used for quantitative and qualitative analysis of naringenin, nobiletin, MH, narirutin, naringin, hesperidin and neohesperidin in the plasma, hippocampus and cortex following oral administration of ZQ extract at a dosage of 20 g/kg. This method was successfully applied to the pharmacokinetic study of ZQ *in vivo* due to the good simplicity, efficiency and repeatability. Additionally, it was also confirmed that naringenin, nobiletin, narirutin, naringin, hesperidin, neohesperidin, and especially MH might be the main effective substances in ZQ extract that provide the antidepressant effect of ZQ *in vivo*. Therefore, these findings offer some guidance for the clinical use of ZQ.

## Author Contributions

PR, XH, JW, and XZ designed experiments, wrote the manuscript, and acted as guarantor. XZ, LH, and QX performed chromatographic analysis. XZ, JL, WZ, and YG carried out animal experiments. PR, XZ, and LH analyzed experimental results.

## Conflict of Interest Statement

The authors declare that the research was conducted in the absence of any commercial or financial relationships that could be construed as a potential conflict of interest.
